# Dietary Intake of High-Protein Foods and Other Major Foods in Meat-Eaters, Poultry-Eaters, Fish-Eaters, Vegetarians, and Vegans in UK Biobank

**DOI:** 10.3390/nu9121317

**Published:** 2017-12-02

**Authors:** Kathryn E. Bradbury, Tammy Y. N. Tong, Timothy J. Key

**Affiliations:** Cancer Epidemiology Unit, Nuffield Department of Population Health, Old Road Campus, Roosevelt Drive, University of Oxford, Oxford OX3 7LF, UK; tammy.tong@ndph.ox.ac.uk (T.Y.N.T.); tim.key@ndph.ox.ac.uk (T.J.K.)

**Keywords:** UK Biobank, vegetarian, vegan, dietary intakes, food groups

## Abstract

Vegetarian diets are defined by the absence of meat and fish, but differences in the intake of other foods between meat-eaters and low or non-meat eaters are also important to document. We examined intakes of high-protein foods (meat, poultry, fish, legumes, nuts, vegetarian protein alternatives, dairy products, and eggs) and other major food groups (fruit, vegetables, bread, pasta, rice, snack foods, and beverages) in regular meat-eaters, low meat-eaters, poultry-eaters, fish-eaters, vegetarians, and vegans of white ethnicity participating in UK Biobank who had completed at least one web-based 24-h dietary assessment (*n* = 199,944). In regular meat-eaters, around 25% of total energy came from meat, fish, dairy and plant milk, cheese, yogurt, and eggs. In vegetarians, around 20% of energy came from dairy and plant milk, cheese, yoghurt, eggs, legumes, nuts, and vegetarian protein alternatives, and in vegans around 15% came from plant milk, legumes, vegetarian alternatives, and nuts. Low and non-meat eaters had higher intakes of fruit and vegetables and lower intakes of roast or fried potatoes compared to regular meat-eaters. The differences in the intakes of meat, plant-based high-protein foods, and other foods between meat-eaters and low and non-meat eaters in UK Biobank may contribute to differences in health outcomes.

## 1. Introduction

Vegetarian diets are defined by the absence of meat and fish; similarly, vegan diets are defined by the absence of all animal foods—meat, fish, dairy products, and eggs. Previous studies have observed lower rates of ischaemic heart disease [1] and some cancers [2,3] in vegetarians. Comparisons of nutrient intakes between meat-eaters and vegetarians and vegans have been previously documented [4,5,6,7,8]. Two large studies [8,9] and some smaller studies have described differences in the intakes of many foods between meat-eaters and low and non-meat eating diet groups [10,11,12]; here we report the first comprehensive data on food intakes among people of different diet groups from a large (*n* = 199,944) cohort in the UK.

Nutritional epidemiological studies have modeled the effects of replacing red and processed meat with substitutes such as chicken, fish, low-fat dairy products, nuts, vegetables and potatoes on health outcomes including coronary heart disease [13,14,15], stroke [16], cancer [17], and mortality [18,19], in cohorts where the vast majority of participants eat red meat. However, there is a paucity of information on the actual food intakes of low- and non-meat eaters and this information is needed to inform realistic substitution analyses of meat alternatives.

The UK Biobank is a large contemporary prospective cohort where participants were invited up to five times to complete a web-based 24-h dietary assessment that assesses actual intake of foods and drinks consumed in the previous 24-h. The objective of this paper is to compare intakes of major protein food sources includingred and processed meat, poultry, fish, legumes and pulses, vegetarian protein alternatives (soy burgers/sausages; tofu; Quorn (a mycoprotein-based food); other vegetarian alternatives), nuts, cheese, yogurt, dairy milk, plant milk, and eggs, and other main food groups including fruit, vegetables, pasta, rice, bread, snack foods, and beverages, in white adult men and women of a range of diet groups—regular meat-eaters, low meat-eaters, poultry-eaters, fish-eaters, vegetarians, and vegans—who participated in UK Biobank and completed at least one web-based 24-hour dietary assessment.

## 2. Materials and Methods

### 2.1. Study Participants

UK Biobank is a prospective cohort study of half a million men and women aged 40–69 years recruited from across the UK between 2006 and 2010 [20]. The UK Biobank protocol is available online [21]. Participants were identified from National Health Service patient registers and invited to attend a local assessment center to participate in UK Biobank. Permission for access to patient records for recruitment was approved by the National Information Governance Board for Health and Social Care in England and Wales, and the Community Health Index Advisory Group in Scotland. At the assessment center participants completed a touchscreen questionnaire, which collected information on sociodemographic characteristics and diet, lifestyle, reproductive, and environmental factors. The touchscreen questionnaire is available on the UK Biobank website [22] UK Biobank has ethical approval from the North West Multi-centre Research Ethics Committee. All participants gave informed consent to participate in UK Biobank and be followed up, using a signature capture device. In April 2009, an enhancement was added to the UK Biobank protocol, whereby a web-based 24-h dietary assessment tool was added to the recruitment visit [23]. The last 70,000 participants who were recruited into UK Biobank completed this 24-h dietary assessment. In addition, the web-based 24-h dietary assessment was emailed out once every 3–4 months for a total of four times from between February 2011 and June 2012, to participants who provided UK Biobank with an email address [24]. A large sub-sample of participants (*n* = 175,402) completed at least one online 24-h dietary assessment during this period. The web-based 24-h dietary assessment asks about the consumption of up to 206 types of foods and 32 types of drinks during the previous 24 h. Compared to an interviewer-administered 24-h recall completed on the same day, Spearman’s correlation coefficients for estimates of the majority of nutrients ranged between 0.5–0.9 (mean of 0.6) for the two methods [25]. Participants who identified as of white ethnicity in the touchscreen questionnaire were eligible for inclusion in this study (94% of participants). For most other ethnic groups, the numbers of non-meat eaters were small. The exception was British Indians, among whom there were 1409 meat-eaters and 432 vegetarians who participated in UK Biobank and completed at least one web-based 24-h dietary assessment. However, we were not confident that the dietary assessment tool would capture all the relevant food items likely to be consumed by these groups, and therefore we did not include them in this study.

### 2.2. Diet Group Classification

Participants were classified as regular meat-eaters, low meat-eaters, poultry-eaters, fish-eaters, vegetarians, or vegans on the basis of their responses to the dietary questions in the touchscreen questionnaire completed at recruitment. The dietary questions in the touchscreen questionnaire generally asked about frequency of consumption of major food groups. Relevant questions used to classify participants into diet group were the questions on the frequency of consumption of beef, lamb, pork, processed meat, chicken or poultry, oily fish, other fish, and the question on avoidance of foods including eggs or foods containing eggs, and dairy products. Participants were classified as regular meat eaters if they reported consuming red meat (the sum of beef, lamb, and pork) more than three times a week, low meat-eaters were those that reported consuming red meat, but three or fewer times a week. Poultry-eaters were those that did not report consuming red or processed meat, but did report consuming poultry, and fish-eaters were those that did not report consuming red or processed meat, or poultry, but did report eating at least one of oily fish or other fish. Vegetarians were those that did not report eating red or processed meat, or poultry, or oily or other fish; vegans additionally reported never eating eggs or foods containing eggs, ordairy products. Participants of unknown diet group were excluded from this analysis (participants who we were not able to classify into a diet group because they answered “Do not know” or “Prefer not to answer” to one of the necessary questions used to define diet groups).

### 2.3. Mean Intake of Food Groups

To estimate the mean intakes of foods and food groups from the 24-h dietary assessments, we used the steering file for the web-based 24-h dietary assessment that lists the portion size of each food item, and, where appropriate, we grouped together food items that constitute a food group. For example, to get the mean intakes of non-oily fish in the touchscreen questionnaire, we calculated the sum (in grams) of the following items from the 24-h dietary assessment: white fish, battered fish, breaded fish, and tinned tuna. For each participant who completed a 24-h dietary assessment, the serving size in grams for each relevant food item was multiplied by the frequency reported in the 24-h dietary assessment. To calculate the percentage energy from foods that are major protein sources we used the steering file, which lists the foodcodes that correspond to each food item, to calculate mean energy in kJ from each relevant food item. Generally, we assigned one whole food item in the 24-h dietary assessment tool to a food group, e.g., the food item ‘apple’ was assigned to the fruit food group. However, the food item ‘eggs in sandwiches’ was disaggregated into ‘egg’ and ‘mayonnaise’ based on standard recipes, and the egg component was assigned to the egg food group. Details of the food items from the web-based dietary assessment tool that were assigned to each food group are given in Supplementary Tables S1 and S2.

### 2.4. Statistical Analysis

We excluded 24-h dietary assessment records where the energy intake was greater than our pre-specified sex-specific acceptable limit (20,000 kJ for men and 18,000 kJ for women). Participant characteristics were described by diet group. Participants who completed more than one web-based 24-h dietary assessment had their records averaged. Mean intakes of foods that are major sources of protein, and other major food groups, from the web-based 24-h dietary assessments were generated by diet group, adjusted for age (continuous). We also repeated the analysis standardising the diets to a 2000 kcal (8368 kJ) per day diet. To do this, for each participant we took the intake of each food item from each 24-h assessment and divided it by their energy intake (in kJ) from that 24-h assessment, and multiplied it by 2000 kcal (8368 kJ). Analysis of covariance was used to test for heterogeneity between the diet groups, with age at recruitment included as a covariate. If there was significant heterogeneity, post hoc pairwise comparisons were used to test for differences between the diet groups, with Bonferroni correction for multiple comparisons. Results reported in the text as differences between two groups are significant pairwise comparisons after correction for multiple comparisons. We also calculated relative mean consumption of broader food groups for each of the low- and non-meat eating groups compared to regular meat-eaters. To do this, for each broad food group we took the ratio of the mean (g) intake for each low- and non-meat eating group to the mean intake (g) of the regular meat-eating group, after adjustment for age. Results for men and women are presented separately. Stata version 14.1 (Stata Corp LP, College Station, TX, USA) was used for all analyses. All *p*-values were two-sided and *p* < 0.05 was considered significant. Statistical code is available on request by emailing the corresponding author.

## 3. Results

Of the 200,541 participants who identified as of white ethnicity, and completed at least one web-based 24-h dietary assessment tool with an energy intake below the sex-specific acceptable limit, 199,944 could be assigned to a diet group based on their answers to the dietary questions in the touchscreen questionnaire. In total, there were 90,742 regular meat-eaters, 97,124 low meat-eaters, 2259 poultry-eaters, 5701 fish-eaters, 3870 vegetarians, and 248 vegans.

Participant characteristics are shown in Table 1 (men) and Table 2 (women). Non-meat eaters (fish-eaters, vegetarians, and vegans) were younger than meat-eaters. Regular and low meat-eaters were less likely to have a college or university degree, and had a higher Body Mass Index (BMI) and body fat percentage on average. Poultry-eaters and non-meat eaters were more likely to have a high level of physical activity compared to regular meat-eaters.

Intakes of foods (in grams and as a percent of energy) that are major sources of protein are shown in Table 3 (men) and Table 4 (women). For regular meat-eaters, meat and fish supplied 12.6% of the total energy intake. Dairy and plant milks supplied approximately another 5%, and cheese, yoghurt, and eggs supplied around 6% of total energy intake. In vegetarians, dairy and plant milks supplied approximately 3–5% of total energy, cheese, yoghurt and eggs supplied 8–9%, and legumes, vegetarian protein alternatives, and nuts supplied 6–7%. In vegans, plant milk supplied 2–3% of total energy, and legumes, vegetarian protein alternatives, and nuts supplied approximately 10%.

Intakes of other major food groups (in grams) are shown in Table 5 (men) and Table 6 (women). There were significant differences in the intakes of most food groups across the diet groups. Low meat-eaters, poultry-eaters, fish-eaters, vegetarians and vegans had higher fruit and vegetable intakes, and lower roast or fried potato intakes compared to regular meat-eaters. Fish-eaters, vegetarians and vegans had higher intakes of wholemeal pasta and brown rice and lower intakes of white bread compared to regular meat-eaters. Among men, fish-eaters, vegetarians and vegans had significantly higher intakes of wholemeal bread, and among women, fish-eaters and vegetarians had higher intakes of wholemeal bread compared to regular meat-eaters. Vegetarians also had significantly higher intakes of white pasta, breakfast cereal and pure fruit/juice and significantly lower intakes or sugar-sweetened beverages compared to regular meat-eaters. Among men, fish-eaters and vegans had significantly lower intakes of coffee, and among women, fish-eaters and vegetarians had lower intakes of coffee, compared to regular meat-eaters. For both men and women, fish-eaters and vegetarians had significantly lower intakes of alcoholic drinks, and in men vegans also had lower intake of alcoholic drinks compared to regular meat-eaters.

**Figure 1:**
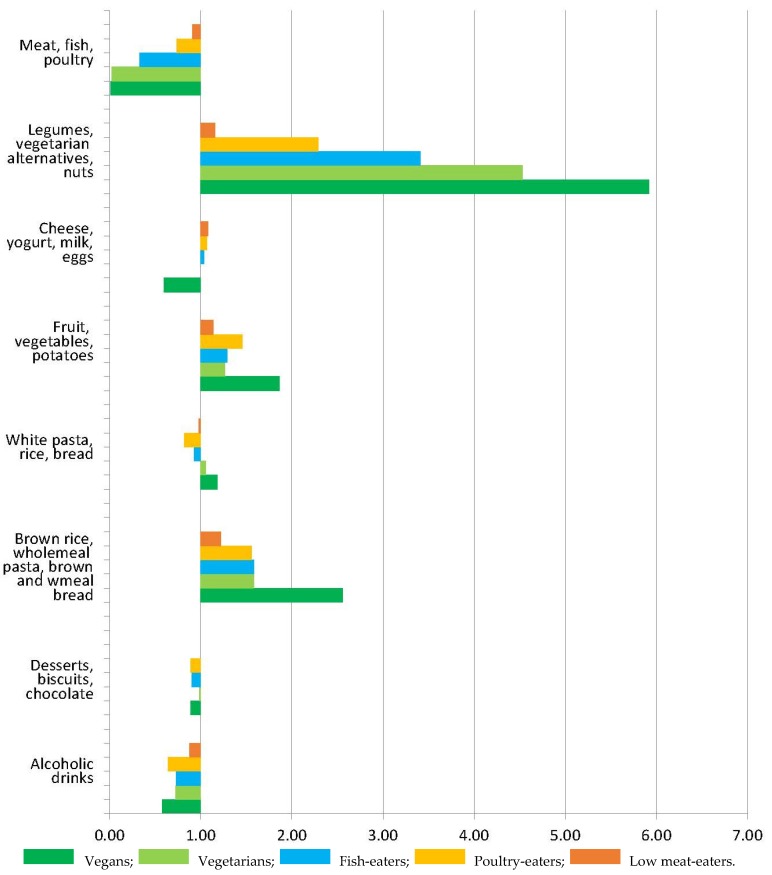
Relative consumption of food groups (g) in low meat-eaters, poultry-eaters, fish-eaters, vegetarian, and vegan men compared to regular meat-eaters. The mean consumption relative to regular meat-eaters (1.00) is shown for each food group after adjustment for age.

**Figure 2:**
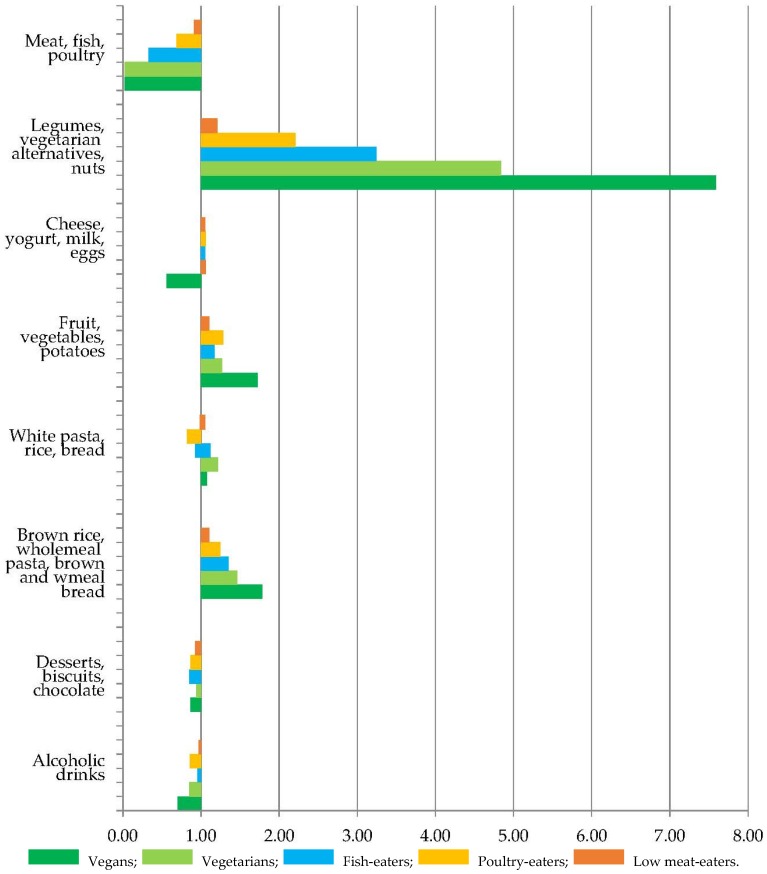
Relative consumption of food groups (g) in low meat-eaters, poultry-eaters, fish-eaters, vegetarian, and vegan women compared to regular meat-eaters. The mean consumption relative to regular meat-eaters (1.00) is shown for each food group after adjustment for age.

Figure 1 (men) and Figure 2 (women) show the relative mean consumption of broad food groups in the low- and non-meat eating groups, compared to the regular meat-eaters. Mean intakes (g) of the sum of legumes, vegetarian protein alternatives, and nuts were over 3-fold higher in fish-eaters, vegetarians, and vegans compared to regular meat-eaters. Mean intakes (g) of the sum of brown and wholemeal bread, wholemeal pasta, and brown rice were over 1.5 times higher in fish-eaters, vegetarians and vegans compared to regular meat-eaters.

## 4. Discussion

In this paper, we describe mean intakes of major food groups in six diet groups within UK Biobank. We found that regular meat-eaters consumed about a quarter of their total energy intake from high-protein source foods (meat, fish, milk, cheese, yoghurt, eggs, legumes, nuts, and vegetarian alternatives). In contrast vegetarians consumed about 20% and vegans around 15% of total energy from these high-protein source foods. We also found that vegetarians and vegans consumed more fruit, vegetables, wholemeal pasta and brown rice, and less fried or roast potatoes, than regular meat-eaters. Additionally, vegetarians consumed significantly more breakfast cereals, and pure fruit/vegetable juice and less sugar-sweetened beverages compared to regular meat-eaters.

The Adventist Health Study-2 is a cohort of Seventh-day Adventist men and women from the North America with large numbers of low and non-meat eating participants. Similar to our study, the Adventist Health Study-2 documented higher intakes of legumes, meat analogues, soyabeans and tofu, tree nuts, and soya milk and lower intakes of soda, fried potatoes, coffee and alcoholic beverages in the low and non-meat eating groups [9]. The Adventist Health Study-2 observed lower intakes of tea in their vegan, vegetarian and semi-vegetarian groups [9], whereas in UK Biobank there were no significant differences in tea intake between vegetarian and vegans and meat-eaters. This difference could be explained by the primary motivation underlying the choice to reduce or avoid meat in the low- and non-meat eating groups in the two cohorts; low- and non- meat eaters in the Adventist Health Study-2 are likely to be motivated by religious beliefs to avoid or reduce meat, alcohol, and caffeine containing drinks (including tea) [26]. We do not have information in UK Biobank on the religion of participants or on the underlying reasons and motivations for choosing a vegetarian or other low- or non-meat eating diet, however it is likely that there are a number of contributing motivators, including animal welfare, environmental concerns and taste preferences [27]. Nevertheless, in general we found dietary patterns in the non- and low meat-eaters participating in UK Biobank similar to those in the Adventist Health Study-2. Also in agreement with our results EPIC-Oxford a British cohort with large numbers of non-meat eaters, observed higher intakes of cheese, vegetables and fruit and lower intakes of dairy milk in vegetarians (*n* = 16,081) compared to non-vegetarians (*n* = 31,173), but did not examine intake of other food groups [28]. The NutriNet-Santé study in France has also compared food intakes between their meat-eaters (*n* = 90,664), vegetarian (*n* = 2370), and vegan (*n* = 789) participants. Similar to our findings, they found higher intakes of fruit, vegetables, legumes, and nuts, vegetarian patties, and textured soy protein products, and lower intakes of alcoholic beverages in the vegetarians and vegans; in addition, vegans had lower intakes of sugary drinks [8]. Another American study with a small number of vegetarians found higher intakes of legumes, fruit and vegetables, cereals, pasta, and wine in vegetarians (*n* = 120) compared to meat-eaters (*n* = 12,543) [12]. A study of different diet groups in Belgium reported higher fruit intakes in vegetarians (*n* = 573) and vegans (*n* = 104), and higher intakes of whole grains in vegetarians compared to meat-eaters (*n* = 155) [10]. A very small study of Finnish vegans (*n* = 22) and non-vegetarians (*n* = 15) similarly found higher intakes of legumes, and tofu and soy flour in vegans compared to non-vegetarians [11].

Strengths of this study include the large total sample size and the detailed collection of information on the actual food and beverages consumed by the participants. The web-based 24-h dietary assessment questionnaire allowed participants to select from over 200 food items and over 30 beverages to record dietary intake during the previous 24 h. Plant-based meat alternatives are represented in the questionnaire, including ‘soy burgers/sausages’, ‘tofu’, ‘Quorn’ (a mycoprotein-based food), and ‘other vegetarian alternative’. However, it is possible that the dietary assessment tool may not have captured all the plant based food items that non-meat eaters consume. A limitation is that for each item in the 24-h dietary assessments, participants were asked to select how many portions they consumed, for example the specified portion for apples was one apple. However, for some items such as pasta and rice, participants were asked how many ‘servings’ they consumed. It is possible that some low and non-meat eaters consume larger portions of foods such as pasta and rice than meat-eaters, and if so, we would not have captured this unless they selected multiple servings to reflect their larger portion. In addition, we classified participants into diet groups based on their answers to the dietary questions on the touchscreen questionnaire administered at recruitment. It is possible that some participants changed diet group by the time they completed one or more of the web-based 24-h dietary assessments. While some degree of misclassification is unavoidable, the very low mean intakes of red and processed meat reported in the 24-h dietary assessments by our non-meat eating groups suggest the diet groups stayed largely stable over the time period of the study. We averaged intakes of major food groups within each diet group, but it is possible that there are distinct dietary patterns within diet groups. However, it was beyond the scope of this report to explore different dietary patterns within a diet group. Finally, although the dietary assessment was recent—2010 to 2012—the range and availability of vegetarian and vegan food products is evolving and the food choices of low and non-meat eaters are likely to be changing.

There is a lot of interest in the impact of meat on health, for example in relation to colorectal cancer [29]. A vegetarian diet is defined by the absence of meat and fish, and previous studies have modeled the replacement of red meat with poultry and fish [13,15,16,17,18,19], dairy products [13,16,19], eggs [16], nuts [13,16,19], legumes [13,16,19], vegetables and potatoes [14], and wholegrains [19] in relation to disease outcomes. Our results can be used to inform realistic modeling scenarios. We found that that in this population, rather than eating an equivalent amount of energy from any other single high-protein food group, low and non-meat eaters are likely to consume more of a wide variety of different foods, including fruit and vegetables, brown rice, wholemeal bread, breakfast cereals, and pure fruit and vegetable juice. There were many differences in food intake between regular meat-eaters and low and non-meat eaters, regardless of whether we expressed daily food intake as percentage of total energy, absolute grams, or grams standardized to a 2000 kcal diet.

In addition to health implications, food choices also have environmental implications. The production of plant-based foods is generally associated with lower greenhouse gas emissions than the production of animal food products [30] and we have previously shown in EPIC-Oxford that the estimated greenhouse gas emissions from the production of foods consumed by fish-eaters, vegetarians, and vegans are lower than from the production of foods consumed by meat-eaters [31].

## 5. Conclusions

In conclusion, we examined the intakes of main protein source foods and other major food groups in a large contemporary cohort of white British people of different diet groups: regular meat-eaters, low meat-eaters, poultry-eaters, fish-eaters, vegetarians and vegans. Our results suggest that low- and non-meat eaters only partially replace red and processed meat with other high-protein source foods, and that they eat more of a variety of plant-based foods. These findings can be used to inform realistic dietary substitution analyses. As well as the absence or reduction of meat, the other differences in food choices between low- and non-meat eating diets may contribute to differences in health outcomes between diet groups.

## Figures and Tables

**Table 1:** Characteristics of men in UK Biobank by diet group.

	Diet Group ^1^					
	Regular Meat-Eaters	Low Meat-Eaters	Poultry-Eaters	Fish-Eaters	Vegetarian	Vegan
Characteristic	*n* = 51,144	*n* = 35,235	*n* = 508	*n* = 1560	*n* = 1243	*n* = 102
Age (years)	56.8 (8.0)	57.2 (7.9)	57.0 (8.1)	54.1 (7.8)	52.6 (7.7)	53.1 (7.5)
College or University degree ^2^	42.2 (21,557)	44.7 (15,764)	51.4 (261)	64.6 (1007)	63.9 (794)	47.1 (48)
Current smokers ^2^	10.2 (5238)	7.5 (2648)	5.7 (29)	7.2 (112)	8.3 (103)	8.8 (9)
Height (cm)	176.6 (6.7)	176.5 (6.6)	176.8 (7.0)	177.3 (6.7)	177.2 (6.5)	177.5 (7.3)
BMI (kg/m^2^)	27.8 (4.2)	27.1 (3.9)	25.7 (3.9)	25.5 (3.5)	25.7 (3.8)	24.7 (3.3)
Body fat (%)	25.2 (5.7)	24.3 (5.6)	22.1 (6.1)	21.8 (5.8)	22.3 (5.7)	21.0 (5.7)
High level of physical activity ^2,3^	18.0 (9213)	18.7 (6572)	24.8 (126)	20.7 (323)	19.3 (240)	22.6 (23)

Values are mean (SD) unless otherwise stated. ^1^ High meat-eaters are those that reported eating the total of red and processed meat three or more times a week, low meat-eaters reported eating the total of red and processed meat fewer than three times a week, poultry-eaters reported eating poultry but no red or processed meat, fish-eaters reported eating fish but no red or processed meat or poultry, vegetarians did not report eating red or processed meat, poultry or fish, and vegans additionally reported never eating eggs or foods containing eggs or dairy products; ^2^ Values are percent (number); ^3^ Defined as 50 or more excess metabolic equivalent hours per week.

**Table 2:** Characteristics of women in UK Biobank by diet group.

	Diet Group ^1^					
	Regular Meat-Eaters	Low Meat-Eaters	Poultry-Eaters	Fish-Eaters	Vegetarian	Vegan
Characteristic	*n* = 39,598	*n* = 61,889	*n* = 1751	*n* = 4141	*n* = 2627	*n* = 146
Age (years)	55.9 (7.9)	56.0 (7.7)	56.0 (7.9)	53.9 (7.8)	52.5 (7.8)	53.5 (8.2)
College or University degree ^2^	37.9 (14,992)	40.7 (25,190)	47.2 (826)	59.0 (2444)	58.4 (1533)	58.9 (86)
Current smokers ^2^	7.0 (2758)	6.6 (4057)	5.5 (96)	5.9 (243)	5.8 (151)	7.5 (11)
Height (cm)	163.3 (6.1)	163.4 (6.2)	163.3 (6.2)	164.2 (6.2)	163.9 (6.0)	163.4 (6.1)
BMI (kg/m^2^)	27.2 (5.2)	26.3 (4.8)	24.8 (4.5)	24.8 (4.4)	25.0 (4.8)	24.1 (4.9)
Body fat (%)	36.8 (6.8)	35.6 (6.8)	33.0 (7.2)	33.0 (7.0)	33.3 (7.4)	31.1 (7.8)
High level of physical activity ^2,3^	14.6 (5775)	15.8 (9792)	20.7 (362)	19.0 (787)	18.5 (485)	21.2 (31)

Values are mean (SD) unless otherwise stated. ^1^ High meat-eaters are those that reported eating the total of red and processed meat three or more times a week, low meat-eaters reported eating the total of red and processed meat fewer than three times a week, poultry-eaters reported eating poultry but no red or processed meat, fish-eaters reported eating fish but no red or processed meat or poultry, vegetarians did not report eating red or processed meat, poultry or fish, and vegans additionally reported never eating eggs or foods containing eggs or dairy products; ^2^ Values are percent (number); ^3^ Defined as 50 or more excess metabolic equivalent hours per week.

**Table 3:** Intakes of major protein source foods in men in UK Biobank by diet group.

Total Energy Intake	Diet Group ^1^						*p* for Difference
	Regular Meat-Eaters	Low Meat-Eaters	Poultry-Eaters	Fish-Eaters	Vegetarian	Vegan	
	*n* = 51,144	*n* = 35,235	*n* = 508	*n* = 1560	*n* = 1243	*n* = 102	
	2310	2168	2164	2246	2259	2037	<0.0001
**Major protein source food**							
Red meat							
g/day	50.4	36.6	4.5	0.8	0.8	0.1	<0.0001
g/2000 kcal/day ^2^	45.0	34.9	4.1	1.1	1.0	0.2	<0.0001
% of energy	4.7	3.6	0.4	0.1	0.1	0.0	<0.0001
Processed meat							
g/day	25.2	15.7	3.2	0.4	-	-	<0.0001
g/2000 kcal/day ^2^	22.1	14.6	2.8	0.6	-	-	<0.0001
% of energy	2.5	1.6	0.3	0.1	-	-	<0.0001
Poultry							
g/day	36.0	35.9	37.9	-	-	-	<0.0001
g/2000 kcal/day ^2^	32.5	34.4	36.0	-	-	-	<0.0001
% of energy	3.2	3.3	3.4	-	-	-	<0.0001
Oily fish							
g/day	9.6	12.5	25.7	19.1	1.2	0.6	<0.0001
g/2000 kcal/day ^2^	8.6	11.9	24.0	18.0	1.3	0.6	<0.0001
% of energy	1.0	1.3	2.7	2.1	0.1	0.1	<0.0001
Non-oily fish							
g/day	16.1	16.6	23.5	21.8	0.6	0.4	<0.0001
g/2000 kcal/day ^2^	14.7	16.2	23.7	21.1	0.9	0.8	<0.0001
% of energy	1.2	1.2	1.7	1.5	-	-	<0.0001
Legumes/pulses							
g/day	15.5	15.3	20.7	27	33.1	52.1	<0.0001
g/2000 kcal/day ^2^	13.8	14.5	20.2	24.9	32	51.2	<0.0001
% of energy	0.6	0.7	1.0	1.3	1.6	2.7	<0.0001
Vegetarian protein alternatives							
g/day	1.2	2.8	16.7	38.4	54.1	56.3	<0.0001
g/2000 kcal/day ^2^	1.1	2.6	16.3	33.6	48.2	52.8	<0.0001
% of energy	0.1	0.2	1.1	2.6	3.4	4.0	<0.0001
Nuts							
g/day	5.5	6.2	9.1	8.7	9.0	14.5	<0.0001
g/2000 kcal/day ^2^	4.4	5.2	7.7	7.3	7.3	10.3	<0.0001
% of energy	1.1	1.3	1.9	1.9	1.9	2.7	<0.0001
Cheese ^3^							
g/day	17.7	17.1	18.0	25.9	30.7	6.1	<0.0001
g/2000 kcal/day ^2^	15.4	15.7	16.4	23.2	27.6	6.1	<0.0001
% of calories	2.8	2.9	2.8	4.2	5.0	1.1	<0.0001
Yogurt ^3^							
g/day	33.0	39.3	47.5	40.4	36.4	30.5	<0.0001
g/2000 kcal/day ^2^	30.4	38.5	47.5	38.8	34.7	33.2	<0.0001
% of energy	1.2	1.5	1.8	1.6	1.4	1.3	<0.0001
Dairy milk							
g/day	221	219	189	181	165	14	<0.0001
g/2000 kcal/day ^2^	202	214	183	172	155	33	<0.0001
% of energy	4.7	4.8	4.0	3.9	2.5	0.7	<0.0001
Plant milk							
g/day	4	7	22	26	33	86	<0.0001
g/2000 kcal/day ^2^	4	7	23	25	32	86	<0.0001
% of energy	0.1	0.1	0.4	0.5	0.6	1.8	<0.0001
Eggs							
g/day	22.1	19.8	21.2	25.3	23.5	2.9	<0.0001
g/2000 kcal/day ^2^	19.3	18.0	19.5	22.4	20.7	4.4	<0.0001
% of energy	1.7	1.6	1.7	2	1.8	0.4	<0.0001
**Total % of calories from**							
Meat and fish	12.6	11.0	8.5	3.8	0.2	0.1	
Dairy and plant milk	4.8	4.9	4.4	4.4	3.1	2.5	
Cheese, yogurt ^3^ and eggs	5.7	6.0	6.3	7.8	8.2	2.8	
Legumes, nuts and vegetarian alternatives	1.8	2.2	4.0	5.8	6.9	9.4	
Total major protein source foods	24.9	24.1	23.2	21.8	18.4	14.8	

All values are adjusted for age. ^1^ High meat-eaters are those that reported eating the total of red and processed meat three or more times a week, low meat-eaters reported eating the total of red and processed meat fewer than three times a week, poultry-eaters reported eating poultry but no red or processed meat, fish-eaters reported eating fish but no red or processed meat or poultry, vegetarians did not report eating red or processed meat, or poultry or fish, and vegans additionally reported never eating eggs or foods containing eggs and dairy products; ^2^ Intakes are standardised to 2000 kcal/day (8368 kJ/day); ^3^ The questionnaire did not specify whether cheese or yogurt was dairy or plant-based (i.e., soy). Vegans who selected cheese or yogurt are likely to have consumed plant-based products.

**Table 4:** Intakes of major protein source foods in women in UK Biobank by diet group.

Total Energy Intake	Diet Group ^1^						*p* for Difference
	Regular Meat-Eaters	Low Meat-Eaters	Poultry-Eaters	Fish-Eaters	Vegetarian	Vegan	
	*n* = 39,598	*n* = 61,889	*n* = 1751	*n* = 4141	*n* = 2627	*n* = 146	
	1997	1889	1842	1913	1901	1842	<0.0001
**Major protein source food**							
Red meat							
g/day	44.4	31.1	4.3	1.4	0.6	1.3	<0.0001
g/2000 kcal/day ^2^	46.3	33.8	4.9	1.5	0.7	0.9	<0.0001
% of energy	4.7	3.4	0.5	0.2	0.1	0.1	<0.0001
Processed meat							
g/day	18.7	12.7	2.9	0.6	-	0.1	<0.0001
g/2000 kcal/day ^2^	19.3	13.8	3.3	0.7	-	0.0	<0.0001
% of energy	2.1	1.5	0.3	0.1	-	-	<0.0001
Poultry							
g/day	35.6	35.3	33.8	0.9	-	0.2	<0.0001
g/2000 kcal/day ^2^	37.1	39.2	39	1.1	-	1.0	<0.0001
% of energy	3.5	3.7	3.6	0.1	-	0.1	<0.0001
Oily fish							
g/day	10.1	12.2	18.1	18.0	1.0	0.8	<0.0001
g/2000 kcal/day ^2^	10.4	13.3	20.3	19.3	1.1	0.8	<0.0001
% of energy	1.1	1.5	2.3	2.2	0.1	0.1	<0.0001
Non-oily fish							
g/day	14.8	15.5	18.4	18.7	0.8	0.5	<0.0001
g/2000 kcal/day ^2^	15.7	17.6	21.7	20.7	0.9	0.5	<0.0001
% of energy	1.2	1.3	1.5	1.4	0.1	-	<0.0001
Legumes/pulses							
g/day	11.9	12.7	17.5	20.9	28.7	44.8	<0.0001
g/2000 kcal/day ^2^	12.4	14	19.3	22.8	31.5	51.9	<0.0001
% of energy	0.6	0.7	1.0	1.2	1.6	2.8	<0.0001
Vegetarian protein alternatives							
g/day	1.4	2.6	12.8	26.7	43.8	64.7	<0.0001
g/2000 kcal/day ^2^	1.4	2.8	13.7	28.3	47.4	71.9	<0.0001
% of energy	0.1	0.2	0.1	2.0	3.1	5.0	<0.0001
Nuts							
g/day	4.4	4.9	6.7	7.2	7.7	12.4	<0.0001
g/2000 kcal/day ^2^	4.1	4.8	6.6	6.9	7.7	12.0	<0.0001
% of energy	1.0	1.2	1.6	1.7	1.9	2.9	<0.0001
Cheese ^3^							
g/day	16.4	16.5	18.4	24.2	28.7	7.2	<0.0001
g/2000 kcal/day ^2^	16.66	17.6	20.1	25.7	30.8	8.2	<0.0001
% of energy	2.9	3.0	3.3	4.3	5.1	1.4	<0.0001
Yogurt ^3^							
g/day	45.9	52.4	59.9	54.9	54.1	24.5	<0.0001
g/2000 kcal/day ^2^	49.4	59.3	70.5	61.1	61.4	27.3	<0.0001
% of energy	1.9	2.3	2.7	2.4	2.4	1.0	<0.0001
Dairy milk							
g/day	214	202	171	179	165	22	<0.0001
g/2000 kcal/day ^2^	226	226	195	196	185	26	<0.0001
% of energy	5.0	5.0	4.3	4.4	4.1	0.6	<0.0001
Plant milk							
g/day	7.5	12.3	28.2	26.7	37.2	96.8	<0.0001
g/2000 kcal/day ^2^	8.1	13.8	31.6	29.6	42.1	118	<0.0001
% of energy	0.2	0.3	0.6	0.6	0.8	2.4	<0.0001
Eggs							
g/day	20.4	19.4	23.1	22.5	20.1	1.1	<0.0001
g/2000 kcal/day ^2^	20.7	20.6	24.1	23.6	21.4	1.3	<0.0001
% of energy	1.9	1.9	2.2	2.2	1.9	0.1	<0.0001
**Total % of calories from**							
Meat and fish	12.6	11.4	8.2	3.9	0.3	0.2	
Dairy and plant milk	5.2	5.3	4.9	5.0	4.9	3.0	
Cheese, yogurt ^3^ and eggs	6.7	7.2	8.2	8.9	9.4	2.5	
Legumes, nuts and vegetarian alternatives	1.7	2.1	2.7	4.9	6.6	10.7	
Total major protein source foods	26.2	26.0	24.0	22.7	21.2	16.4	

All values are adjusted for age. ^1^ High meat-eaters are those that reported eating the total of red and processed meat three or more times a week, low meat-eaters reported eating the total of red and processed meat fewer than three times a week, poultry-eaters reported eating poultry but no red or processed meat, fish-eaters reported eating fish but no red or processed meat or poultry, vegetarians did not report eating red or processed meat, or poultry or fish, and vegans additionally reported never eating eggs or foods containing eggs and dairy products; ^2^ Intakes (g) are standardised to 2000 kcal/day (8368 kJ/day); ^3^ The questionnaire did not specify whether cheese or yogurt was dairy or plant-based (i.e., soy). Vegans who selected cheese or yogurt are likely to have consumed plant-based products.

**Table 5:** Intakes of other food groups in White British men in UK Biobank by diet group.

Food Group	Diet Group ^1^						*p* for Difference
	Regular Meat-Eaters	Low Meat-Eaters	Poultry-Eaters	Fish-Eaters	Vegetarian	Vegan	
	*n* = 51,382	*n* = 35,235	*n* = 508	*n* = 1560	*n* = 1243	*n* = 102	
Fruit							
g/day	165	192	260	217	200	270	<0.0001
g/2000 kcal/day ^2^	159	200	275	219	204	299	<0.0001
Vegetables							
g/day	170	179	237	232	237	359	<0.0001
g/2000 kcal/day ^2^	153	171	227	217	219	360	<0.0001
Potatoes—baked/boiled or mashed							
g/day	55	53	57	56	55	60	0.0005
g/2000 kcal/day ^2^	50	51	55	53	51	58	0.23
Potatoes—roast or fried							
g/day	39	30	19	22	28	19	<0.0001
g/2000 kcal/day ^2^	31	25	16	18	23	16	<0.0001
White pasta							
g/day	23	25	21	30	31	30	<0.0001
g/2000 kcal/day ^2^	21	24	21	28	30	34	<0.0001
Wholemeal pasta							
g/day	4	6	12	11	9	20	<0.0001
g/2000 kcal/day ^2^	3	6	11	10	8	19	<0.0001
White rice							
g/day	15	15	15	14	15	18	0.8764
g/2000 kcal/day ^2^	14	15	15	14	15	23	0.0006
Brown rice							
g/day	3	3	6	7	6	23	<0.0001
g/2000 kcal/day ^2^	2	3	6	6	6	20	<0.0001
Sliced white bread							
g/day	23	16	9	10	14	10	<0.0001
g/2000 kcal/day ^2^	20	15	9	9	13	8	<0.0001
Sliced brown bread							
g/day	19	19	16	20	21	18	0.0237
g/2000 kcal/day ^2^	17	18	15	19	19	20	0.0031
Sliced wholemeal bread							
g/day	21	24	33	34	34	46	<0.0001
g/2000 kcal/day ^2^	19	23	32	30	32	46	<0.0001
Other bread							
g/day	12	13	14	18	18	17	<0.0001
g/2000 kcal/day ^2^	10	11	12	16	16	18	<0.0001
Porridge							
g/day	26	34	55	39	30	48	<0.0001
g/2000 kcal/day ^2^	24	34	57	38	28	56	<0.0001
Breakfast cereals							
g/day	25	29	31	37	34	33	<0.0001
g/2000 kcal/day ^2^	22	27	29	34	31	32	<0.0001
Ice-cream ^3^							
g/day	15	13	10	11	11	5	<0.0001
g/2000 kcal/day ^2^	12	12	9	9	10	6	<0.0001
Milk-based desserts							
g/day	12	12	9	9	10	5	<0.0001
g/2000 kcal/day ^2^	10	10	9	8	9	2	0.0005
Soya desserts							
g/day	0	1	1	2	2	12	<0.0001
g/2000 kcal/day ^2^	0	0	1	1	2	13	<0.0001
Other desserts							
g/day	21	20	17	20	22	15	<0.0001
g/2000 kcal/day ^2^	18	18	15	18	19	13	0.04
Chocolate							
g/day	10	9	9	8	8	5	<0.0001
g/2000 kcal/day ^2^	8	8	8	7	7	6	<0.0001
Biscuits/cereal bars							
g/day	18	16	15	16	17	18	<0.0001
g/2000 kcal/day ^2^	15	15	14	14	15	16	0.0062
Crisps							
g/day	10	8	6	6	8	9	<0.0001
g/2000 kcal/day ^2^	8	7	5	5	7	8	<0.0001
Pure fruit/vegetable juice							
g/day	110	116	141	136	144	139	<0.0001
g/2000 kcal/day ^2^	99	110	134	125	134	144	<0.0001
Cordial/squash							
g/day	52	48	51	43	37	56	<0.0001
g/2000 kcal/day ^2^	46	45	49	39	35	87	0.0004
Sugar sweetened beverages							
g/day	50	37	27	24	31	25	<0.0001
g/2000 kcal/day ^2^	44	35	22	20	30	52	<0.0001
Tea							
g/day	486	481	498	528	505	532	<0.0001
g/2000 kcal/day ^2^	465	489	516	513	497	622	0.005
Coffee							
g/day	347	334	287	293	324	245	<0.0001
g/2000 kcal/day ^2^	331	358	292	281	314	-^4^	<0.0001
White wine							
g/day	36	36	32	35	23	10	<0.0001
g/2000 kcal/day ^2^	31	33	29	31	21	10	<0.0001
Red wine							
g/day	74	70	47	70	63	35	<0.0001
g/2000 kcal/day ^2^	64	68	43	63	55	32	<0.0001
Beer/cider							
g/day	325	248	178	206	215	112	<0.0001
g/2000 kcal/day ^2^	271	220	163	173	189	169	<0.0001

All values are adjusted for age. ^1^ High meat-eaters are those that reported eating the total of red and processed meat three or more times a week, low meat-eaters reported eating the total of red and processed meat fewer than three times a week, poultry-eaters reported eating poultry but no red or processed meat, fish-eaters reported eating fish but no red or processed meat or poultry, vegetarians did not report eating red or processed meat, or poultry or fish, and vegans additionally reported never eating eggs or foods containing eggs and dairy products; ^2^ Intakes are standardised to 2000 kcal/day (8368 kJ/day); ^3^ The questionnaire did not specify whether ice-cream was dairy or plant-based (i.e., soy). Vegans who selected ice-cream are likely to have consumed plant-based products; ^4^ Due to small numbers, a valid standardised mean intake could not be calculated.

**Table 6:** Intakes of other food groups in White British women in UK Biobank by diet group.

Food Group	Diet Group ^1^						*p* for Difference
	Regular Meat-Eaters	Low Meat-Eaters	Poultry-Eaters	Fish-Eaters	Vegetarian	Vegan	
	*n* = 39,940	*n* = 61,889	*n* = 1751	*n* = 4141	*n* = 2627	*n* = 146	
Fruit							
g/day	186	205	241	224	221	263	<0.0001
g/2000 kcal/day ^2^	203	240	294	265	263	343	<0.0001
Vegetables							
g/day	214	224	258	270	284	369	<0.0001
g/2000 kcal/day ^2^	224	247	293	296	314	451	<0.0001
Potatoes—baked/boiled or mashed							
g/day	52.3	49	46.6	46.9	51.2	59.7	<0.0001
g/2000 kcal/day ^2^	55.3	54.2	52.8	20.9	56.2	70.6	0.004
Potatoes—roast or fried							
g/day	33.2	26.2	17.9	20.2	19.1	17.6	<0.0001
g/2000 kcal/day ^2^	30.1	24.8	17.3	18.5	18.3	17.2	<0.0001
White pasta							
g/day	25	26	23	28	29	22	0.0001
g/2000 kcal/day ^2^	26	28	26	30	33	26	<0.0001
Wholemeal pasta							
g/day	3	4	6	7	8	14	<0.0001
g/2000 kcal/day ^2^	3	5	7	7	9	15	<0.0001
White rice							
g/day	14	14	13	15	15	15	0.0168
g/2000 kcal/day ^2^	15	15	15	16	17	18	0.2233
Brown rice							
g/day	2	3	5	6	6	12	<0.0001
g/2000 kcal/day ^2^	2	4	6	7	7	15	<0.0001
Sliced white bread							
g/day	13	9	5	6	7	7	<0.0001
g/2000 kcal/day ^2^							
Sliced brown bread							
g/day	14	13	12	13	14	11	<0.0001
g/2000 kcal/day ^2^	15	14	13	14	15	11	<0.0001
Sliced wholemeal bread							
g/day	17	17	18	20	21	20	<0.0001
g/2000 kcal/day ^2^	17	18	20	22	23	25	<0.0001
Other bread							
g/day	12	13	14	16	17	19	<0.0001
g/2000 kcal/day ^2^	12	14	15	17	18	20	<0.0001
Porridge							
g/day	32	38	44	38	38	40	<0.0001
g/2000 kcal/day ^2^	35	43	51	43	44	50	<0.0001
Breakfast cereals							
g/day	23	24	24	28	27	26	<0.0001
g/2000 kcal/day ^2^	23	25	26	29	29	28	<0.0001
Ice-cream ^3^							
g/day	13	11	11	10	11	5	<0.0001
g/2000 kcal/day ^2^	12	11	11	9	11	6	<0.0001
Milk-based desserts							
g/day	11	10	9	8	9	2	<0.0001
g/2000 kcal/day ^2^	11	10	9	7	9	3	<0.0001
Soya desserts							
g/day	1	1	2	1	2	16	<0.0001
g/2000 kcal/day ^2^	1	1	1	2	3	17	<0.0001
Other desserts							
g/day	21	19	16	18	19	16	<0.0001
g/2000 kcal/day ^2^	20	19	16	18	19	16	<0.0001
Chocolate							
g/day	9	9	8	8	9	6	<0.0001
g/2000kcal/day ^2^	9	8	8	8	9	7	0.0003
Biscuits/cereal bars							
g/day	15	14	13	14	13	12	<0.0001
g/2000 kcal/day ^2^	15	14	14	14	13	10	<0.0001
Crisps							
g/day	7	6	5	6	6	6	<0.0001
g/2000 kcal/day ^2^	7	6	5	6	6	7	<0.0001
Pure fruit/vegetable juice							
g/day	96	95	94	110	110	127	<0.0001
g/2000 kcal/day ^2^	97	102	105	118	117	132	<0.0001
Cordial/squash							
g/day	44	40	34	35	36	25	<0.0001
g/2000 kcal/day ^2^	44	43	38	38	38	26	<0.0001
Sugar sweetened beverages							
g/day	35	27	22	20	20	6	<0.0001
g/2000 kcal/day ^2^	34	28	23	21	22	7	<0.0001
Tea							
g/day	535	520	529	543	534	545	<0.0001
g/2000 kcal/day ^2^	587	610	661	673	680	671	<0.0001
Coffee							
g/day	322	309	285	293	302	274	<0.0001
g/2000 kcal/day ^2^	377	389	352	354	517	324	0.2566
White wine							
g/day	52	51	43	51	38	28	<0.0001
g/2000 kcal/day ^2^	31	33	29	31	21	10	<0.0001
Red wine							
g/day	51	53	46	53	39	31	<0.0001
g/2000 kcal/day ^2^	48	46	42	47	42	41	0.0028
Beer/cider							
g/day	43	36	30	35	38	39	<0.0001
g/2000 kcal/day ^2^	41	37	32	37	39	33	0.0004

All values are adjusted for age. ^1^ High meat-eaters are those that reported eating the total of red and processed meat three or more times a week, low meat-eaters reported eating the total of red and processed meat fewer than three times a week, poultry-eaters reported eating poultry but no red or processed meat, fish-eaters reported eating fish but no red or processed meat or poultry, vegetarians did not report eating red or processed meat, or poultry or fish, and vegans additionally reported never eating eggs or foods containing eggs and dairy products; ^2^ Intakes are standardised to 2000 kcal/day (8368 kJ/day); ^3^ The questionnaire did not specify whether ice-cream was dairy or plant-based (i.e., soy). Vegans who selected ice-cream are likely to have consumed plant-based products.

## Supplementary Materials

The following are available online at http://www.mdpi.com/2072-6643/9/12/1317/s1, Table S1: Food items within each major protein source food group, Table S2: Food items within each food group.
