# Supplementary material for: Dietary Intake of High-Protein Foods and Other Major Foods in Meat-Eaters, Poultry-Eaters, Fish-Eaters, Vegetarians, and Vegans in UK Biobank
Source: Nutrients. 2017 Dec 2;9(12):1317. doi: 10.3390/nu9121317 (PMC5748767; doi:10.3390/nu9121317)
Supplement: Supplementary file 1 [file nutrients-09-01317-s001.pdf]

Supplementary Table S1: Individual food items within each major protein source food group

Food group	Individual food items
Red meat	beef; lamb; pork
Processed meat	sausage; bacon; ham
Poultry	crumbed chicken; chicken/poultry
Oily fish	oily fish
Non-oily fish	white fish; breaded fish; battered fish; tinned tuna
Legumes/pulses	baked beans; pulses; hummus
Vegetarian protein alternatives	soy burgers/sausages; tofu; Quorn; other vegetarian alternatives
Nuts	salted peanuts; unsalted peanuts; salted other nuts; unsalted other nuts
Cheese	low fat hard cheese; hard cheese; soft cheese; blue cheese; low fat cheese spread; cheese spread; cottage cheese; feta cheese; mozarella cheese; goat cheese; other cheese
Yogurt	full fat yogurt; low fat yogurt
Dairy milk	whole milk; semi-skimmed milk; skimmed milk; powdered milk; goat/sheep milk
Plant milk	soya milk with calcium; soya milk without calcium; rice/oat other vegetable milk
Eggs	whole eggs; omelette; eggs disaggregated from mayonnaise in egg sandwiches

Supplementary Table S2. Food items within each other food group

Food group	Individual items
Fruit	stewed/cooked fruit; prunes; other dried fruit; mixed fruit; apple; banana; berries; cherries; grapefruit; grapes; mango; melon; orange; orange-like small fruits; peach, nectarine; pear; pineapple; plum; other fruit
Vegetables	mixed vegetables; vegetable pieces; coleslaw; mixed side salad; avocado; broad beans; green beans; beetroot; broccoli; butternut squash; cabbage; carrots; cauliflower; celery; courgette; cucumber; garlic; leeks; lettuce; mushrooms; onion; parsnip; peas; peppers; spinach; sprouts; sweetcorn; sweet potatoes; tomatoes, fresh; tomatoes, cooked or tinned; turnip or swede; watercress; other vegetables
Potatoes - baked/boiled or mashed	potatoes (baked, boiled); potatoes (mashed)
Potatoes - fried/roast	potatoes (fried, chips, wedges, roast)
White pasta	white pasta

Wholemeal pasta	wholemeal pasta
White rice	white rice
Brown rice	brown rice
Sliced white bread	sliced white bread
Sliced brown bread	sliced brown bread
Sliced wholemeal bread	sliced wholemeal bread
Other bread	naan bread; garlic bread; crackers, crispbread, rice cakes, corn cakes; oat cakes; other bread
Porridge	porridge, hot oat cereal (e.g. Ready Brek)
Breakfast cereal	muesli; sweetened oat crunch type cereal; other sweetened cereals; plain cereals; bran cereals; wholewheat cereals; other cereals
Ice-cream	ice-cream
Milk desserts	custard, rice pudding, semolina pudding, blancmange; other milk based desserts (e.g. mousse, tiramisu, crème caramel); cheesecake
Soya desserts	soya ice cream, soya yogurt, other soya dessert
Other desserts	fruit cake; cake; doughnuts; sponge pudding; other dessert
Chocolate	chocolate bars, white chocolate, milk chocolate, dark chocolate, chocolate sweets
Biscuits/cereal bars	chocolate covered biscuits; chocolate biscuits; sweet biscuits; cereal bars; other sweet snack
Pure fruit/vegetable juice	pure orange juice; pure grapefruit juice; other pure fruit/vegetable juice
Cordial/squash	fruit drinks, $J_{2}0$, squash or cordial
Sugar sweetened beverages	carbonated (fizzy) drinks
Tea	standard tea (e.g. Tetley, PG Tips, Assam, Darjeeling); rooibos/redbush tea (e.g. Tick Tock); green tea; herbal or fruit tea (infusion); other tea or infusion
Coffee	instant; filter/americano/cafetiere; cappuccino; latte; espresso; other coffee drinks
White wine	white wine
Red wine	red wine
Beer/cider	beer lager or cider
